# Therapeutic Effects of VEGF Gene-Transfected BMSCs Transplantation on Thin Endometrium in the Rat Model

**DOI:** 10.1155/2018/3069741

**Published:** 2018-10-30

**Authors:** Zhao Jing, Yan Yi, Huang Xi, Lun-Quan Sun, Li Yanping

**Affiliations:** ^1^Reproductive Medicine Center, Xiangya Hospital, Central South University, Changsha, Hunan, China; ^2^Center for Molecular Medicine, Xiangya Hospital, Central South University, Hunan, China

## Abstract

**Objective:**

Bone mesenchymal stem cells (BMSCs) transplantation has a therapeutic effect on the thin endometrium in animal researches and clinical trials. The present study aims at assessing whether transplantation of VEGF-transfected BMSCs (VEGF-BMSCs) have a better therapeutic effect on endometrial regeneration and endometrial receptivity compared with BMSCs therapy alone.

**Methods:**

Sprague-Dawley (SD) rats were used in the study. Thin endometrium model was established with 95% ethanol injection into uterine. VEGF-BMSCs or BMSCs was transplanted via tail vein IV injection. Endometrial thickness, morphology, and pinopodes were assessed by hematoxylin and eosin (HE) staining and scanning electron microscope (SEM). The proteins and mRNAs expressions of markers for endometrial cells and endometrial receptivity were measured after treatment. The fertility testing was done to assess the embryo implantation efficiency.

**Results:**

VEGF-BMSCs transplantation significantly increased endometrial thickness compared with the BMSCs group and the control group. There was no significant difference in endometrial thickness between VEGF-BMSCs group and sham operation group. Importantly, in protein level, expressions of cytokeratin, vitamin, VEGF, LIF, and integrin *α_ν_β*_3_ in VEGF-BMSC group were increased dramatically compared with those of the control group and BMSC group both 4 days and 8 days after stem cells transplantation. Accordingly, mRNA expression of LIF and integrin *α*_ν_ *β*_3_ was significantly upregulated compared with those of the control group and BMSC group both 4 and 8 days after treatment. The pinopodes were developed better in the VEGF-BMSCs group and the sham operation group compared with BMSCs group and the control group. The number of embryo implantation is largest in the sham operation group, followed by VEGF-BMSCs group, BMSCs group, and the control group.

**Conclusions:**

Transplantation of VEGF gene-transfected BMSCs may be a better therapeutic treatment for thin endometrium than stem cell therapy alone.

## 1. Introduction

A thin endometrium often means impaired endometrial receptivity, which has been well recognized as a critical factor in implantation failure [1–4]. At present, there was no uniform definition for “thin endometrium”. A number of studies suggested that a minimal endometrial thickness of 6 mm is required for embryo implantation [5]. Several treatments, such as estrogen, aspirin, pentoxifylline, and endometrial injury, have been tried to improve the regeneration of the endometrium. However, improvement was not very obvious with the available treatments [6–8]. It is still a big challenge to find a useful treatment for thin endometrium [9].

Until now, the most promising treatment for thin endometrium or Asherman syndrome (AS) seems to be the stem-cell therapy. There have been many animal and human reports about the treatment effect of stem cells transplantation on the thin endometrium. A number of animal experiments showed that the thin endometrium was significantly improved after stem cells transplantation [10–13]. Clinical reports showed that infertile women with thin endometrium or AS had a significant increase in endometrial thickness after stem cells treatment and enhanced pregnancy rates [14–17].

It was reported that thin endometrium pathophysiologic features were as follows: increased flow impedance of radial arteries has a detrimental effect on the glandular epithelium, leading to a decreased level of vascular endothelial growth factor (VEGF). At last, poor vascular development and impaired blood flow of the endometrium were caused [18]. VEGF was an angiogenesis factor and play a critical role in angiogenesis via branching of old blood vessels or sprouting of new blood vessels. Recently, stem cells transplantation has been discussed as a treatment for thin endometrium. So, the present study aimed at investigating whether the VEGF gene-transfected BMSCs (VEGF-BMSCs) have a better effect on the thin endometrium.

## 2. Materials and Methods

### 2.1. Animals

Seven–nine weeks Sprague-Dawley (SD) rats weighing 150–250 g were used in the experiments. All animal experiments were performed in strict line with the “National Institutes of Health Guide for the Care and Use of Laboratory Animals”. The present study was reviewed and approved by the Institutional Review Board and the Ethics Committee of Xiangya Hospital, Central South University.

### 2.2. BMSCs' Collection and Culture

Bone marrow was aspirated and prepared from the femurs and tibia of an adult, male/female, SD rats. The bone marrow was digested, cultured, and filtered according to a previous study [19]. When reaching at least 80% confluence, the stem cells were collected with 0.05% trypsin-EDTA (Gibco). BMSCs were cultured at a density of 3000 to 6000/cm^2^, and the fourth to sixth passages BMSCs were used for transfection and transplantation.

### 2.3. Plasmid Preparation and Transfection of Rat BMSC

Human VEGF_165_ was integrated into the pCR3.1 plasmid, which was published previously. 1 *μ*g of plasmid DNA of hVEGF_165_ was used for transfection per 100,000 cells, and the empty pCR3.1 combined with 3 *μ*L of metafectene was used as control group per 100,000 cells. These cells were transferred to BMSCs culture medium 6 hours later and were kept overnight. Before transplantation, 5^∗^10^6^ of the stem cells was suspended in saline at a concentration of 50,000 cells/*μ*L.

### 2.4. Groups

Standardized laboratory conditions, including air-condition rooms and plenty of water and food, were applied to keep the rats. According to our preliminary study, a thin endometrium rat model was built by injecting anhydrous ethanol into the uterus cavity of rats [20]. One hundred rats were divided into four groups randomly, which included the control group (iv-injected saline into tail vein 6–8 hours after modeling, *n* = 25), BMSC group (iv-injected BMSCs into tail vein 6–8 hours after modeling, *n* = 25), VEGF-BMSC group (in-injected VEGF-BMSCs into tail vein 6–8 hours after modeling, *n* = 25), and sham operation group (operation without modeling, *n* = 25).

For scanning electron microscopy (SEM) and fertility testing, rats were anesthetized and killed with overdose 10% chloral hydrate (1.125 g/kg) at 4 days (*n* = 10) and 9 days (*n* = 10) after the appearance of vaginal plugs. The rats were anesthetized and killed with overdose 10% chloral hydrate (1.125 g/kg) at three estrus cycles after BMSCs treatment. The vaginal smear was observed to determine the estrous cycles. The uteri were excised after the rats were sacrificed. For further research, uteri of rats were sectioned and stored in liquid nitrogen and/or formalin.

### 2.5. Hematoxylin and Eosin (HE) Staining

HE staining was performed according to a previous study [21]. The slides with 5 *μ*m thickness were completely covered in xylene twice and rehydrated in a series of ethanol with gradually decreased concentration. The slides were rinsed with deionized demineralized water, dyed in hematoxylin for about 50 seconds, rinsed with deionized demineralized water once again, and at last dyed in eosin for 3 seconds. The slides of the endometrium were dehydrated with a series of ethanol in xylene and mounted with Permount mounting medium after the color reaction. The endometrial morphology and the endometrial thickness were examined and compared between groups.

### 2.6. Immunohistochemistry

For immunohistochemistry, the uterine were stored in 4% paraformaldehyde and embedded in paraffin. Serial slides with about 6 *μ*m were prepared, deparaffinized in xylene, rehydrated with different concentrations ethanol, and rinsed with water. Endogenous peroxidase was blocked with 3% hydrogen peroxidase. Slides were treated with chondroitin ABC lyase (0.15 U/mL) and blocked with 10% normal goat serum for 1 hour. Expressions of cytokeratin, vimentin, integrin *α_ν_β*_3_, and leukemia inhibitory factor (LIF) proteins were performed by incubating slides of rat uteri with rabbit polyclonal antibodies against cytokeratin, vimentin, integrin *α*_ν_ *β*_3_, and LIF overnight at 4°C. Slides were incubated with secondary antibodies at 1 : 3000 dilution followed by DAB solution for 1 hour. Then, slides were briefly stained with hematoxylin solution (15 seconds) (Gill no. 3; Sigma) and evaluated by a microscope (Nikon).

### 2.7. Western Blotting

Total protein was extracted from uterine tissues according to the protocol, and the concentration of protein was confirmed with a Precision Red Assay (Cytoskeleton Inc.). Equal amounts of protein were stained with loading dye and separated with 12% SDS-PAGE. Protein was then transferred to polyvinylidene difluoride (PVDF) membrane and was incubated with 5% bovine serum albumin (BSA). The membrane was exposed to anti-VEGF antibody (1 : 500), anti-cytokeratin antibody (1 : 500), anti-vimentin antibody (1 : 500), anti-integrin*α_ν_* antibody (1 : 300), anti-integrin*β*_3_ antibody (1 : 300), and anti-LIF antibody (1:300) overnight. The blots were washed with tris-buffer saline and incubated with secondary anti-rabbit IgG (1 : 3000; Cell Signaling Technology) for 1 hour at room temperature. The protein content was detected using SuperSignal West Pico (Pierce Biotechnology). Anti-*β*-actin antiserum (Sigma) was used as an internal standard between groups.

### 2.8. Real-Time PCR

Total RNA was collected from the harvested uteri by the use of an extraction reagent (Trizol; Gibco) and was dissolved in water treated with diethylpyrocarbonate, and the concentration was measured by a spectrophotometer (UV-1601). In order to determine a constant expression of a housekeeping gene in the RNA extractions, the real-time PCR of GAPDH was also performed. Any possible DNA contamination was removed with DNase I (0.2 U/*μ*L; Ambion). Quantitative PCR reaction was performed with Biosystems 7500 Fast Real-Time PCR system and Taqman probes. Quantitative expression level was analyzed using the 2ΔΔCt method. The PCR probe sets used are as follows:

VEGF: sense 5′- CGA CAG AAG GGG AGC AGA AAG-3′, and antisense 5′-GCA AGT ACG TTC GTT TAA CTC-3′;

LIF: sense 5′- GTC AAC TGG CTC AAC TCA ACG-3′, and antisense 5′- CTG GCA GCC CAA CTT CTT C-3′;

Integrin *α_ν_*: sense 5′- GTC AGC CCA GTC GTG TCT TAC A-3′, and antisense 5′- GGG CTT GAA ACT CCT CTT ATC TCA-3′;

Integrin *β*_3_: sense 5′- GTG GAC CGC AAC AAC GCA-3′, and antisense 5′- ACC AAG GTA ACG CCA GGA AT-3′.

### 2.9. Scanning Electron Microscopy (SEM)

The rats were euthanized, and the uteri were dissected and cut open longitudinally. Uteri were submerged in 2.5% glutaraldehyde and kept in this solution for 24 hours. Then the specimens were rinsed several times in phosphate buffer, fixed in 4% osmium phosphate-buffered solution, dehydrated in an acetone solution in distilled water at increasing concentrations, and kept in 100% acetone. Then the samples were dried in a critical point drier with carbon dioxide, mounted and coated with gold, and examined by SEM.

### 2.10. Fertility Testing

Endometrial receptivity was assessed by testing their capacity to receive fertilized ova and retain embryos for pregnancy. The rats were mating at 1 : 1 ratio with mature male rats, euthanized 9 days after the appearance of vaginal plugs, and each uterus was examined for the number of fetuses.

### 2.11. Statistical Analyses

The statistical software SPSS 16.0 (IBM) was applied in the present study. Measurement data were present as mean ± SD. A one-sided *t*-test was used to compare the difference between groups. For Western blot, the density of protein bands was analyzed with Image-J software, and the relative protein level was presented as a ratio of the target protein to *β*-actin. For real-time PCR, the relative mRNA level was obtained by comparing the target mRNA to the GAPDH from the same gel. A *P* level of less than 0.05 (*P* < 005) was considered to be significant.

## 3. Results

### 3.1. BMSC Phenotype

The BMSCs, obtained from rat bone marrow aspirates, were grown in the cultural medium as previously published. FACS analysis showed that CD90 and CD73 were expressed in BMSCs, whereas hematopoietic markers CD45 and CD34 were negative. The BMSCs had the capability to differentiate toward adipocytes and osteoblasts.

### 3.2. Histopathological Observations

Rat in BMSCs and VEGF-BMSCs group had a significantly larger number of endometrial glands, and a significant thicker endometrium compared with that of the control group. The endometrial layer of the VEGF-BMSC group showed a relatively intact structure, with more endometrial glands, capillaries and increased endometrial thickness. The endometrium of the control group was totally damaged, showing extensive coagulation necrosis, cell apoptosis in the nearly whole layer of endometrium and parts of the myometrium layer.

**Figure 1:**
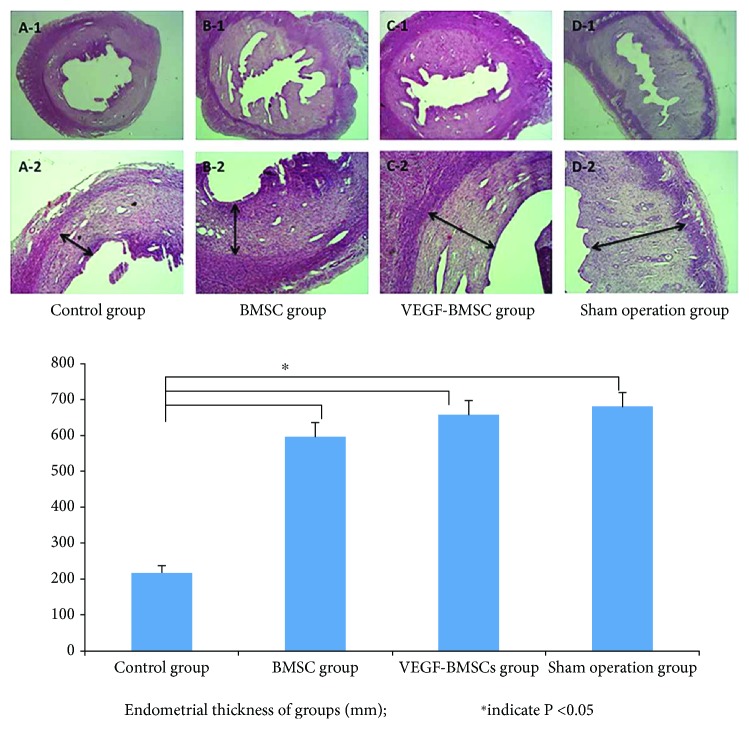
The morphology observation of the endometrium with HE staining (the first line were ×40; the second line were ×200). (A-1,2): control group, (B-1,2): BMSC group, (C-1,2): VEGF-BMSC group, (D-1,2): sham operation group. The bar chart represented the endometrial thickness of groups. The endometrial layer of the VEGF-BMSC group showed a relatively intact structure, with more endometrial glands, capillaries and increased endometrial thickness. The endometrium of the control group was totally damaged, showing extensive coagulation necrosis, cell apoptosis. The VEGF-BMSC group had an obvious thicker endometrial lining compared with that of the control group and the BMSC group, and there was no significant difference between the VEGF-BMSC group and the sham operation group.

The endometrial thickness of the control group, BMSC group, VEGF-BMSC group, and sham operation group were as follows: 218.7 ± 20.6 *μ*m, 598.7 ± 37.7 *μ*m, 658.0 ± 40.0 *μ*m, and 682.3 ± 38.2 *μ*m, respectively. The VEGF-BMSC group had an obvious thicker endometrial lining compared with that of the control group and the BMSC group, and there was no significant difference between the VEGF-BMSC group and the sham operation group (Figure 1).

### 3.3. Protein Expression of VEGF, Cytokeratin, Vimentin, Integrin *α_ν_β*_3_, and LIF

**Figure 2:**
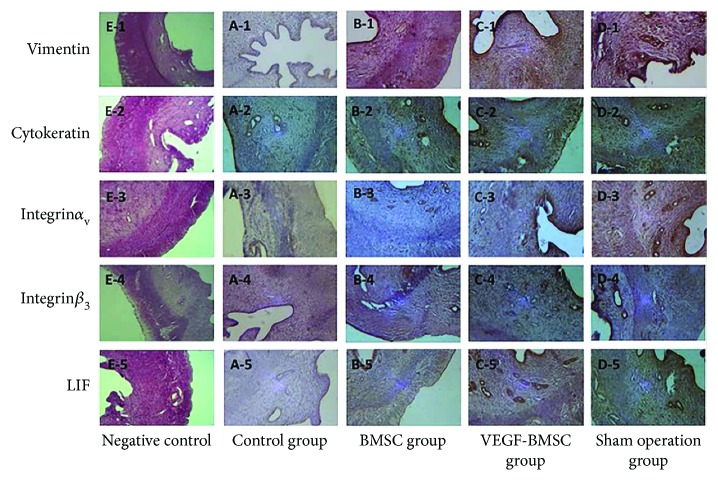
Protein expression of markers for endometrial cells and endometrial receptivity with immunohistochemistry. The expression of cytokeratin, vimentin, integrin*α_ν_β*_3_, and LIF in the VEGF-BMSC group and BMSC group was significantly stronger than those of the control group and were slightly weaker than those of the sham operation group without significance.

Immunohistochemical results demonstrated that cytokeratin, integrin *α*_ν_ *β*_3_, and LIF were mainly expressed in the cytoplasm of the endometrial epithelial cells, and vimentin was mainly localized in the cytoplasm of endometrial stromal cells. The expression of cytokeratin, vimentin, integrin*α*_ν_ *β*_3_, and LIF in the VEGF-BMSC group and BMSC group were significantly stronger than those of the control group and were slightly weaker than those of the sham operation group without significance. (Figure 2, Supplemental Figure 1).

**Figure 3:**
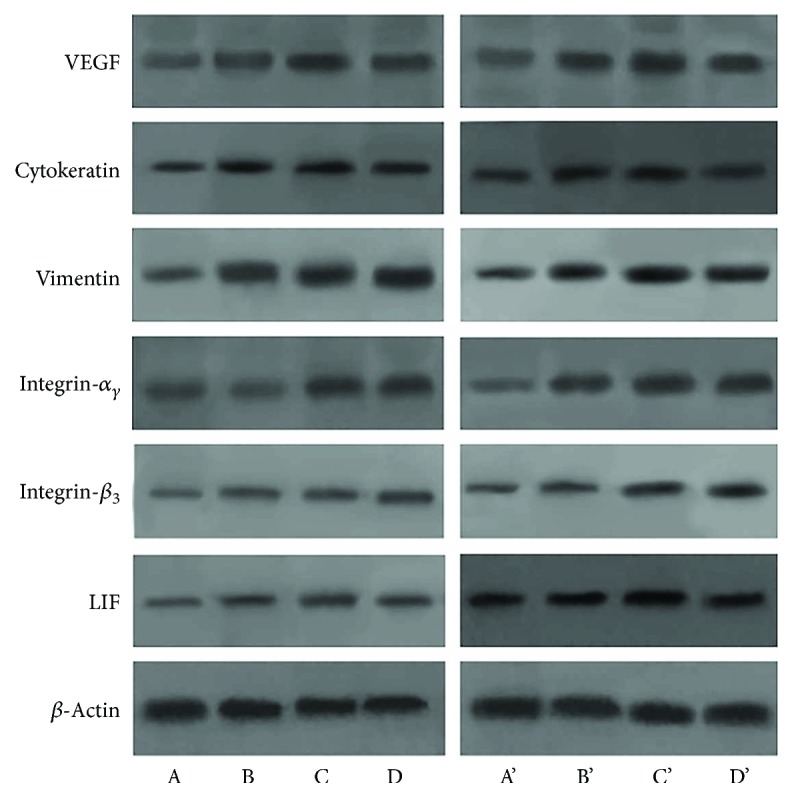
Protein expression of markers for endometrial cells and endometrial receptivity with Western blotting. (A, B, C, D) represent the control group, BMSC group, VEGF-BMSC group, and sham operation group 4 days after treatment, respectively. (A′, B′, C′, D′) mean these four groups 8 days after treatment. Vimentin, integrin *α*_ν_ *β*_3_, and LIF protein expressions were gradually increased in the control, BMSC, VEGF-BMSC, and sham operation groups, respectively. VEGF-BMSC group showed a significantly higher expression of these four proteins compared with those of control group (*P* < 0.05). VEGF expression level was the highest in the VEGF-BMSC group, followed by BMSC group, sham operation group, and control group. The cytokeratin expression level in the VEGF-BMSC group was significantly higher compared with the control group. When compared day 4 with day 8 after stem cells transplantation, there was no significant difference in the expressions of those proteins.

Western Blotting was applied to exam the expression of these proteins in the endometrium 4 days and 8 days after stem cells transplantation. Vimentin, integrin *α_γ_β*_3_, and LIF protein expressions were gradually increased in the control, BMSC, VEGF-BMSC, and sham operation groups, respectively. VEGF-BMSC group showed a significantly higher expression of these four proteins compared with those of control group (*P* < 0.05). VEGF expression level was the highest in the VEGF-BMSC group, followed by BMSC group, sham operation group, and control group. The cytokeratin expression level in the VEGF-BMSC group was slightly higher than that of the BMSC group and sham operation group without a significant difference and was significantly higher compared with the control group. When compared day 4 with day 8 after stem cells transplantation, there was no significant difference in the expressions of those proteins. (Figure 3, Supplemental Figure 2).

### 3.4. mRNA Expression of VEGF, Integrin *α_ν_β*_3_, and LIF

**Figure 4:**
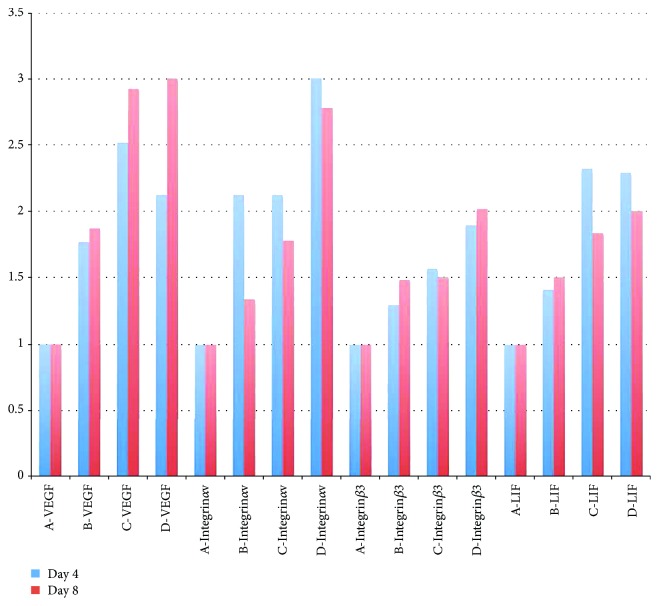
mRNA expressions of endometrial receptivity's markers 4 and 8 days after treatment. (A, B, C, D) represent control group, BMSC group, VEGF-BMSC group, and sham operation group, respectively. The VEGF-BMSC group exhibited a significantly higher expression of VEGF mRNA than the BMSC group and control group. The LIF mRNA expression in the VEGF-BMSC group was significantly higher than that of the BMSC group and control group and was similar with that of the sham operation group. The integrin *α*_ν_ *β*_3_ mRNA expressions in the VEGF-BMSC group and BMSC group were similar, which were significantly higher when compared with the control group.

mRNA expressions of endometrial receptivity's markers 4 and 8 days after treatment are shown in Figure 4 and Supplemental Figure 3. The VEGF-BMSC group exhibited a significantly higher expression of VEGF mRNA than the BMSC group and control group and a similar level with the sham operation group. The LIF mRNA expression in the VEGF-BMSC group was significantly higher than that of the BMSC group and control group and was similar with that of the sham operation group. The integrin *α_ν_ β*_3_ mRNA expressions in the VEGF-BMSC group and BMSC group were similar, which were significantly higher when compared with the control group.

### 3.5. Pinopodes of the Endometrium

**Figure 5:**
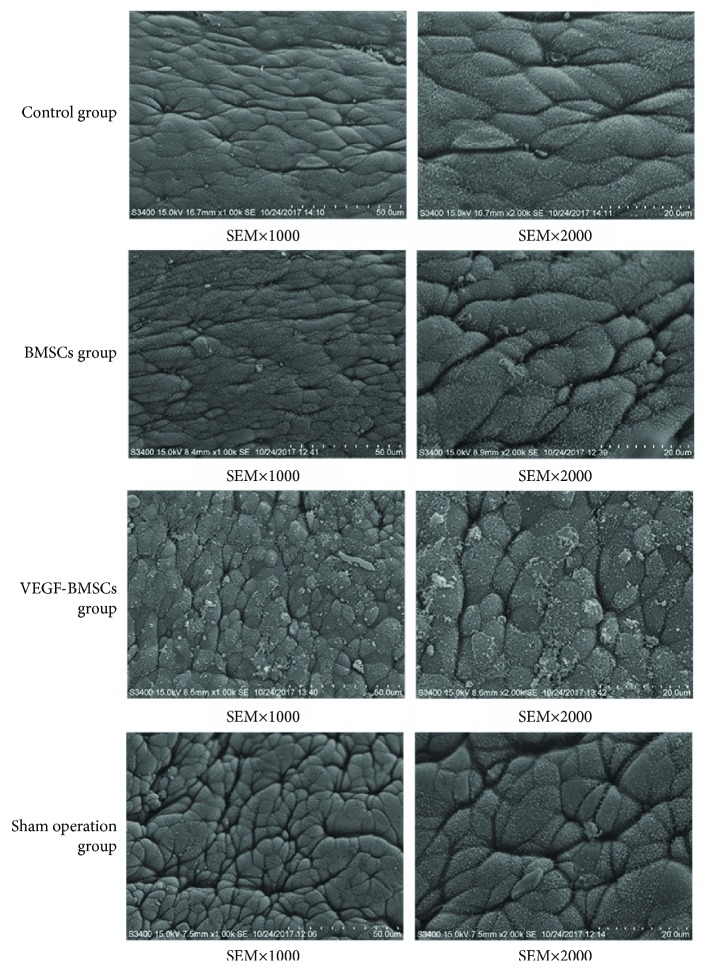
Scanning electron microscopy (×1000, ×2000) of the endometrial surface.

In the control group, the endometrium is thin. The microvilli were abundant, and no developed pinopodes in luminal cells were observed. In the BMSCs group and VEGF-BMSCs group, the microvilli gradually decreased in number and length. Smooth and slender membrane projections were formed from the cell apex and were transformed to pinopodes. We can observe that the pinopodes were developing and some of them had short microvilli in their surface in VEGF-BMSCs group and sham operation group. There were no significant differences between VEGF-BMSCs group and sham operation group in the number of pinopodes (Figure 5).

### 3.6. Embryo Implantation after VEGF-BMSCs/BMSCs Transplantation

**Figure 6:**
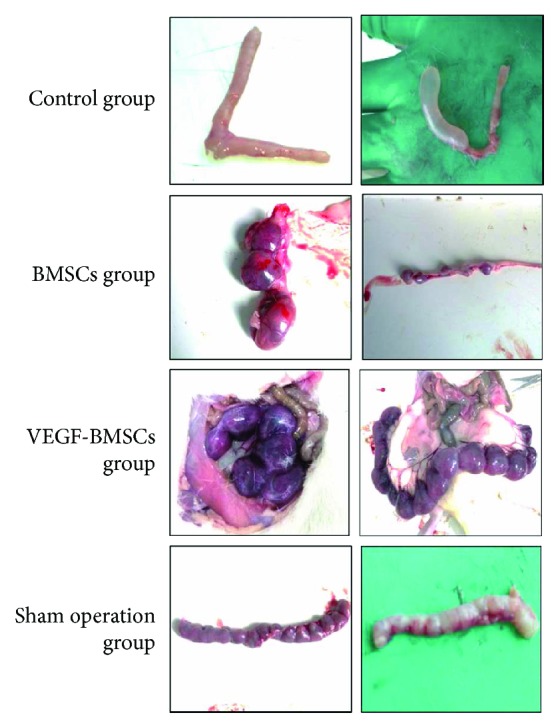
Embryo implantation efficiency of the control group, BMSCs group, VEGF-BMSCs group, and sham operation group.

Embryo implantation efficiency is the best index to assess the therapeutic effect of VEGF-BMSCs/BMSCs transplantation. In order to detect the effect of BMSCs/VEGF-BMSCs on the thin endometrium and endometrial receptivity, we looked at the embryo implantation efficiency in the different groups of rats. The sham operation group showed the greatest embryo implantation efficiency, followed by VEGF-BMSCs group, BMSC group, and control group. Sham operation group showed a significantly increased embryo implantation efficiency compared with BMSCs group and control group, and there was no significant difference between the sham operation group and VEGF-BMSCs group. (Figure 6, Supplemental Figure 4).

## 4. Discussion

There are many experimental studies showing the beneficial effects of VEGF gene-transfected stem cells transplantation on many disorders, such as bone defects, stroke, myocardial infarction, acute kidney injury, and bronchopulmonary dysplasia. [22–25].

At present, there are no reports about the VEGF gene-transfected stem cells transplantation as the treatment for thin endometrium or AS. As we all know, angiogenesis is necessary for the regeneration of endometrium after menstruation and plays a crucial role in the development of endometrial receptivity for successful embryo implantation [26, 27]. A lot of studies have explored the regulation of endometrial angiogenesis and showed that the vascularization of the endometrium was regulated by VEGF, which was expressed in the human endometrium [28–32]. Therefore, the pathophysiology of thin endometrium is damaged angiogenesis and decreased uterine blood flow.

Our previous studies have proved that BMSC transplantation could promote the growth of thin endometrium and improve the endometrial receptivity [12, 13]. It was supposed that VEGF gene-transfected BMSC would be a better therapy for thin endometrium. The present study is the first experiment, which assessed the effect of VEGF-BMSCs transplantation on thin endometrium in a rat model.

In our study, we showed that the rats in the VEGF-BMSC group had a thicker endometrium than those in the control group and BMSC group, and the expression of cytokeratin and vimentin in the VEGF-BMSC group was stronger than that in the control group and BMSC group. What we found indicated that IV infusion of VEGF-BMSCs promotes the regeneration of the endometrial cells and have a stronger therapeutic effect for thin endometrium.

We not only examined the endometrial regeneration but also assessed the endometrial receptivity. The present study found that the mRNA and protein expressions of integrin *α_ν_β*_3_ and LIF have significantly decreased in the thin endometrium rats without BMSC or VEGF-BMSC transplantation, and the BMSC/VEGF-BMSC treatment almost normalized the expression of integrin*α_ν_β*_3_ and LIF. As important regulators of endometrial function, integrin and LIF are considered to be the markers for endometrial receptivity and play a critical role in embryo implantation [33, 34]. Integrins belong to transmembrane glycoproteins family, and integrin *α*_1_ *β*_1_, *α*_4_ *β*_1_, and *α*_ν_ *β*_3_ were found to be coexpressed in endometrium on days 20–24 of the human menstrual cycle [35, 36]. These three integrins have been considered as endometrial receptivity markers, and *α*_ν_ *β*_3_ was found to be important in the course of embryo attachment. LIF receptors are expressed by the blastocyst as well as the endometrium, with maximum expression of LIF mRNA and protein occurring in endometrial epithelium during the implantation window, so it was considered to play an important role in implantation [37].

In addition to the protein marker of endometrial receptivity, we also observed the pinopodes, which are smooth mushroom-like projections that arise from the apical surface of the endometrium and are considered as endometrial receptivity marker [38]. The pinopodes are observed for a short time period, 24 to 48 hours, during implantation in mammals [39], depending on the ovarian hormones, especially progesterone. In the rat endometrium, the appearance of pinopodes clearly demarcates the window of receptivity with a rise in numbers on day 4 of pregnancy, abundance on day 5 [40], and rapid decline on day 6 [38, 41]. The results showed that there were well-developed pinopodes in the sham operation group, BMSCs group, and VEGF-BMSCs group, and there was no pinopode in the control group. The function of pinopodes is not clear. The surfaces of pinopodes may have some receptors for adhesion molecules, which are essential for embryo implantation [42]. A vitro study observed the embryo attachment to endometrial epithelial cell and proposed that pinopode improve the attachment of blastocysts to endometrium during the processes of implantation [43].

Accordingly, the fertility testing demonstrated that embryo implantation efficiency is significantly higher in BMSCs group and VEGF-BMSCs group compared with the control group. The study has confirmed that UC-MSCs can promote endometrial proliferation and recover the endometrial embryo implantation ability [44]. This is consistent with our findings. We found that the VEGF-BMSC transplantation not only promote the endometrial regeneration but also improve the endometrial receptivity, showing a better therapeutic effect compared with BMSCs treatment alone.

Our data, which show a stronger mRNA and protein expressions of cytokeratin, vimentin, integrin *α_ν_β*_3,_ and LIF, indicated that stem cells transplantation might not only bring beneficial effect on the endometrial regeneration but also bring good for endometrial receptivity improvement.

For Asherman's syndrome or thin endometrium, stem cells transplantation seems to be the most promising treatment. Several animal studies showed a significant enhancement of endometrial thickness and receptivity after MSCs transplantation into a rat model of thin endometrium [10–13]. All experimental groups showed an improvement in the fibrosis level and elucidated regenerative capabilities of BMSCs when thin endometrium rats were infused with BMSCs [12, 13]. It was found that transplanted cells could migrate to the injured uterus after intrauterine or tail vein injection. So migration to the injured sites and/or immune-regulation effect was the possible mechanisms of stem cells' effect [10, 11]. Stem cells not only promoted endometrial cell differentiation/proliferation and vascularization [10] but also decreased fibrosis, which ensured tissue repair. At last, stem cell transplantation restored the endometrial function and improved the fertility rate eventually [11].

Stem cells treatment for thin endometrium or AS was also applied in human. The first report of stem cell treatment for human was published in 2011 [14]. Autologous stromal stem cells were transplanted into the endometrial cavity with refractory thin endometrium (3.6 mm) [14]. At last, the endometrium was appropriate for embryo implantation. Another study [16] investigated six women with AS and found that transplantation of autologous BMSCs significantly improved the embryo implantation. Subsequently, Santamaria et al. [15] transplanted autologous CD133+ BMSCs into 11 AS and 5 endometrial atrophies (ET < 5 mm) women and showed endometrial functional restoration two months after cell therapy. Inspiringly, a recent study [17] found that transplantation of autologous menstrual blood-derived stromal cells (menSCs) could increase the endometrial thickness of severe AS; suggested autologous menSCs transplantation might be the most possible choice for severe AS. All of the above studies indicated that stem cells treatment was effective in endometrial regeneration.

The present study firstly explored the effect of VEGF gene-transfected BMSC transplantation on the thin endometrium and showed inspiring results. However, there were also some limitations. Firstly, the present study did not investigate the effect mechanism of VEGF-BMSCs treatment for thin endometrium. Secondly, the safety of VEGF gene-transfected BMSCs transplantation is not clear, so there is a long way to go before clinical application. Thirdly, the present only evaluated the endometrial regeneration and endometrial receptivity and did not observe the embryo implantation and pregnancy after treatment.

## 5. Conclusions

In conclusion, VEGF gene-transfected BMSCs transplantation would be a better therapy for thin endometrium than BMSCs transplantation alone. Further studies, which assess the safety of VEGF-BMSC treatment, the mechanisms of its effect, are needed in the future.

## Data Availability

The data used to support the findings of this study are available from the corresponding author upon request.

## Supplementary Materials

Supplementary Materials Figure S1: AOD of markers for endometrial cells and endometrial receptivity with immunohistochemistry. A, B, C, D represent the control group, BMSC group, VEGF-BMSC group, and sham operation group. Figure S2: relative level of markers for endometrial cells and endometrial receptivity with Western blotting. A, B, C, D represent the control group, BMSC group, VEGF-BMSC group, and sham operation group 4 days and 8 days after treatment. Figure S3: amplification curves, melting curves, and standard curves of PCR. Figure S4: embryo implantation efficiency of the control group, BMSC group, VEGF-BMSC group, and sham operation.
