# Supplementary material for: Therapeutic Effects of VEGF Gene-Transfected BMSCs Transplantation on Thin Endometrium in the Rat Model
Source: Stem Cells Int. 2018 Oct 30;2018:3069741. doi: 10.1155/2018/3069741 (PMC6232792; doi:10.1155/2018/3069741)
Supplement: Supplementary Materials — Figure S1: AOD of markers for endometrial cells and endometrial receptivity with immunohistochemistry. A, B, C, D represent the control group, BMSC group, VEGF-BMSC group, and sham operation group. Figure S2: relative level of markers for endometrial cells and endometrial receptivity with Western blotting. A, B, C, D represent the control group, BMSC group, VEGF-BMSC group, and sham operation group 4 days and 8 days after treatment. Figure S3: amplification curves, melting curves, and standard curves of PCR. Figure S4: embryo implantation efficiency of the control group, BMSC group, VEGF-BMSC group, and sham operation. [file 3069741.f1.docx]

**Supplemental Figures**


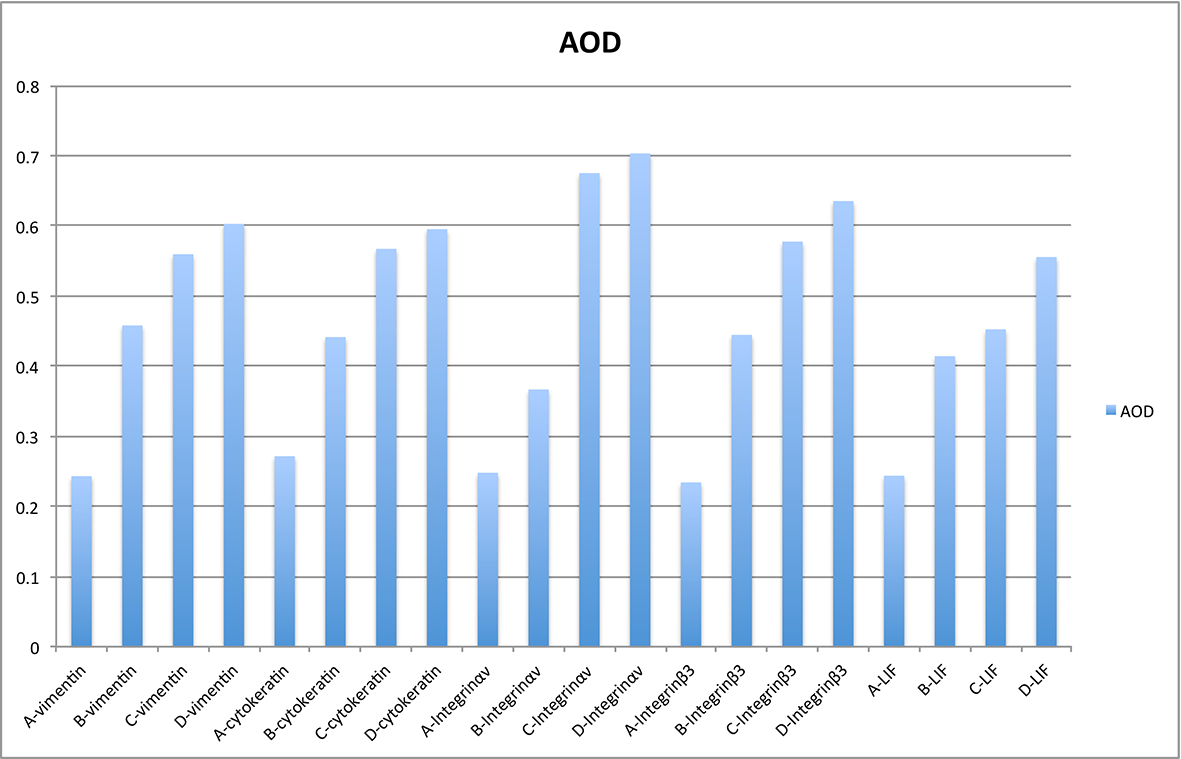


**Supplemental Figure 1.** AOD of markers for endometrial cells and endometrial receptivity with immunohistochemistry. A, B, C, D represent for control group, BMSC group, VEGF-BMSC group, and sham operation group.


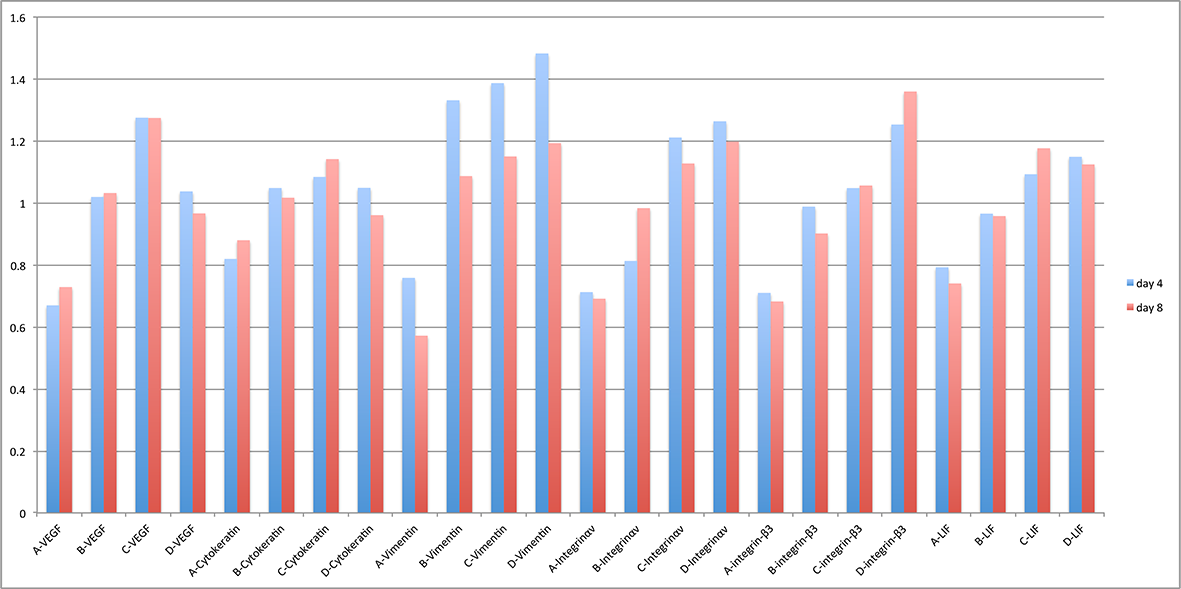


**Supplemental Figure 2.** Relative level of markers for endometrial cells and endometrial receptivity with Western Blotting. A, B, C, D represent for control group, BMSC group, VEGF-BMSC group, and sham operation group 4 days and 8 days after treatment.


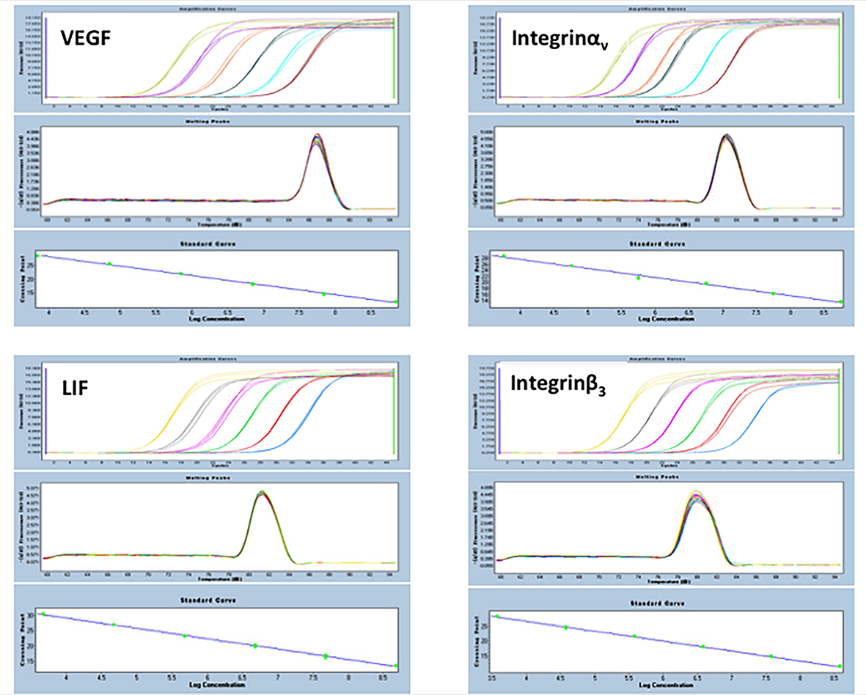


**Supplemental Figure 3.** Amplification curves, Melting curves, and Standard curves of PCR.


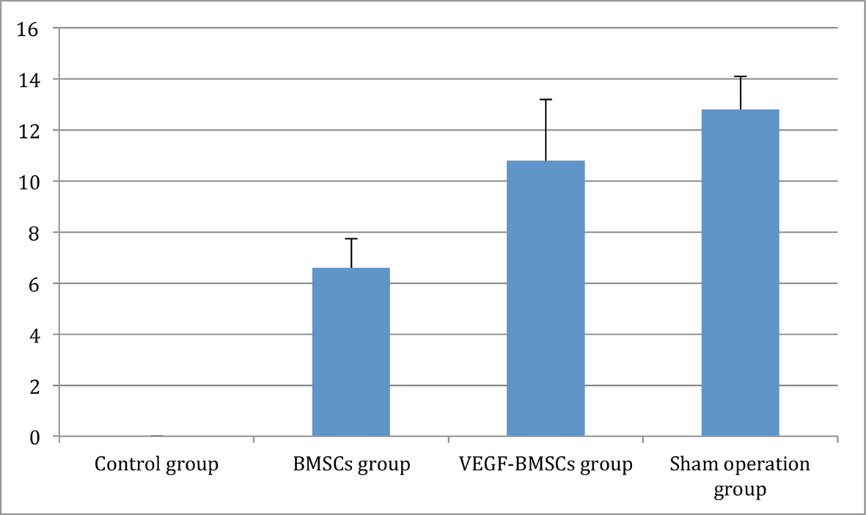


**Supplemental Figure 4** Embryo implantation efficiency of control group, BMSC group, VEGF-BMSC group, and sham operation
